# Serologic evidence of human monocytic and granulocytic ehrlichiosis in israel

**Published:** 2000

**Authors:** Keysary, Waner


### Serologic Evidence of Human Monocytic and Granulocytic Ehrlichiosis in Israel

**To the Editor**: Our article on ehrlichiosis in Israel is one of several reporting serologic evidence of ehrlichiosis outside North America (1-4). Serologic tests were performed for immunoglobulin (IgG) antibodies to ***Ehrlichia chaffeensis***, ***E. canis,*** and human granulocytic ehrlichiosis (HGE) agent to prevent misinterpretation due to cross-reaction. The conclusions meet the criteria published by the American Society for Rickettsiology, in which a confirmed diagnosis of human monocytic ehrlichiosis (HME) is based on a "single serum titer of 256" in a patient with clinically compatible disease (5).

The argument that the same epidemiologic circumstances that exist in the United States have to be prevalent in Israel for the disease to be present is not compelling. Reporting serologic evidence of ehrlichiosis in Israel alerts physicians to the possibility of HME and HGE when they see patients with symptoms compatible with these diseases. We agree that isolation and molecular identification of both agents are essential to confirming the presence of these diseases in Israel.

**Avi Keysary and Trevor Waner**
Israel Institute for Biological Research,
Ness Ziona, Israel

### References

1. Keysary A, Amram L, Keren G, Sthoeger A, Potasman I, Jacob A, et al. Serologic evidence of human monocytic and granulocytic ehrlichiosis in Israel. Emerg Infect Dis 1999;5:775-8.
2. Morais JD, Dawson JE, Greene C, Filipe AR, Galhardas LC, Bacellar F. First European case of ehrlichiosis. Lancet 1991;338:833-4.
3. Bakken JS, Krueth J, Tilden RL, Dumler JS, Kristiansen BE. Serological evidence of human granulocytic ehrlichiosis in Norway. Eur J Microbiol Infect Dis 1996;15:829-32.
4. Christova IS, Dumler JS. Human granulocytic ehrlichiosis in Bulgaria. Am J Trop Med Hyg 1999;60:58-61.
5. Walker DH. Consensus workshop on diagnosis of human ehrlichiosis. Bulletin of the American Society for Rickettsiology 1999;2:1-8.
